# Flammability, Tensile, and Morphological Properties of Oil Palm Empty Fruit Bunches Fiber/Pet Yarn-Reinforced Epoxy Fire Retardant Hybrid Polymer Composites

**DOI:** 10.3390/polym13081282

**Published:** 2021-04-14

**Authors:** M.J. Suriani, Fathin Sakinah Mohd Radzi, R.A. Ilyas, Michal Petrů, S.M. Sapuan, C.M. Ruzaidi

**Affiliations:** 1 Faculty of Ocean Engineering Technology and Informatics, Universiti Malaysia Terengganu, Kuala Nerus 21030, Terengganu, Malaysia; surianimatjusoh@umt.edu.my (M.J.S.); ruzaidi@umt.edu.my (C.M.R.); 2 Marine Materials Research Group, Faculty of Ocean Engineering Technology and Informatics, Universiti Malaysia Terengganu, Kuala Nerus 21030, Terengganu, Malaysia; 3 School of Chemical and Energy Engineering, Faculty of Engineering, Universiti Teknologi Malaysia, UTM Johor Bahru 81310, Johor, Malaysia; 4 Centre for Advanced Composite Materials, Universiti Teknologi Malaysia, UTM Johor Bahru 81310, Johor, Malaysia; 5 Institute for Nanomaterials, Advanced Technologies and Innovation, Technical University of Liberec, Studentská 2, 461 17 Liberec, Czech Republic; michal.petru@tul.cz; 6 Laboratory of Biocomposite Technology, Institute of Tropical Forestry and Forest Products (INTROP), Universiti Putra Malaysia, UPM Serdang 43400, Selangor, Malaysia; sapuan@upm.edu.my; 7 Advanced Engineering Materials and Composites Research Centre (AEMC), Department of Mechanical and Manufacturing Engineering, Faculty of Engineering, Universiti Putra Malaysia, UPM Serdang 43400, Selangor, Malaysia

**Keywords:** OPEFB composite, flammability, fire retardant, hybrid composite, tensile, morphology, pet yarn, epoxy

## Abstract

Oil palm empty fruit bunches (OPEFB) fiber is a natural fiber that possesses many advantages, such as biodegradability, eco-friendly, and renewable nature. The effect of the OPEFB fiber loading reinforced fire retardant epoxy composites on flammability and tensile properties of the polymer biocomposites were investigated. The tests were carried out with four parameters, which were specimen A (constant), specimen B (20% of fiber), specimen C (35% of fiber), and specimen D (50% of fiber). The PET yarn and magnesium hydroxide were used as the reinforcement material and fire retardant agent, respectively. The results were obtained from several tests, which were the horizontal burning test, tensile test, and scanning electron microscopy (SEM). The result for the burning test showed that specimen B exhibited better flammability properties, which had the lowest average burning rate (11.47 mm/min). From the tensile strength, specimen A revealed the highest value of 10.79 N/mm^2^. For the SEM morphological test, increasing defects on the surface ruptured were observed that resulted in decreased tensile properties of the composites. It can be summarized that the flammability and tensile properties of OPEFB fiber reinforced fire retardant epoxy composites were reduced when the fiber volume contents were increased at the optimal loading of 20%, with the values of 11.47 mm/min and 4.29 KPa, respectively.

## 1. Introduction

Nowadays, by increasing worldwide social awareness about the environmental impacts of plastics, the plastic industry has been seeking ecologically friendly materials for their products [1,2,3,4]. The type of renewable source and the new generation of reinforcements and supplements for the polymers that are currently trending is natural fiber [5]. The wide industrial applications and high demand for natural fiber composites have resulted from environmental concern and their low cost, easy processing, low density, good corrosion resistance, and high strength to weight ratios [6,7,8]. Moreover, the natural fiber contains the hydrogen bond and other linkages that give strength and stiffness to the fiber [9,10]. Natural fibers are cellulosic materials with several fibrils that run all along the length of the fiber [11]. Besides, for replacing existing synthetic polymers or glass fibers, the natural fiber is applied in reinforced polymer composites and natural-based resins [12].

A composite material is a material consisting of two or more constituents with complementary features and different natures, this results in a new material having unique and great properties compared with its original constituents [13,14,15,16]. Two basic constituents of a composite material that are essentially insoluble to each other are matrix and reinforcements [17,18]. Moreover, the composition on a macro-scale and combination of two or more materials that differ in size can generally be defined as composite materials [19,20,21,22]. Although they appear to be in synergy (synergistic effect), the components in them still retain their identity [23]. Each component can be physically identified, and there is a boundary between it and other components. In the wood industry, composite materials predominantly contain either wood fibers or wood elements. To bond these elements, a natural or synthetic adhesive is used in this industry [24].

**Figure 1:**
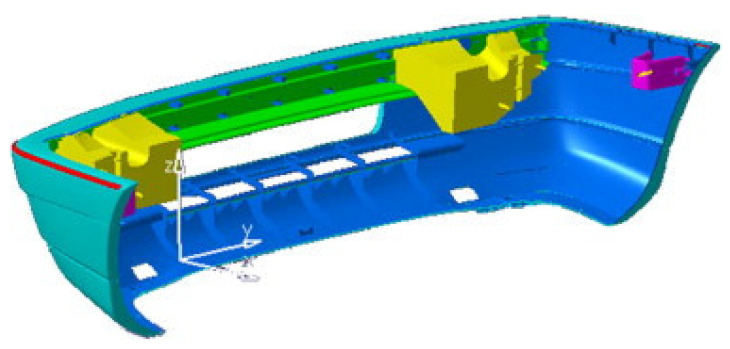
Bumper automotive components. Reproduced with copyright permission from Davoodi et al. [44].

Fiber reinforced polymer matrix composites’ applications are found in households and industries because they have more favorable properties, such as high stiffness, greater strength, better fatigue performance, more corrosion resistance, low thermal expansion, low energy consumption during manufacturing, and non-magnetic properties [25,26,27,28,29,30]. The use of bio-fibers as substitutes for synthetic fibers (i.e., carbon and glass) as fillers in the development of polymer matrix composites has attracted much attention [31]. The properties of oil palm empty fruit bunch bio-fibers (i.e., abundance, low density, low cost, high modulus, harmlessness, and biodegradability) have facilitated the association with polymers to produce composite materials. There is increased awareness about the properties of natural fiber-based epoxy composites to meet engineering requirements [32]. Vijaya Ramnath et al. [33] conducted a study on the evaluation of mechanical properties of abaca–jute–glass fiber reinforced epoxy composite and revealed that abaca fiber had the highest flexural strength compared to jute fiber, with the values of 12.5 and 11.9 MPa, respectively, since its strength increased with improved interfacial adhesion. Besides that, Abaca exhibited more strength when it absorbed moisture. Szolnoki et al. [34] reinforced twill woven hemp fabric with epoxy composites and discovered that the modification of the fabrics led to decreased flammability of the reference matrix composites, characterized with increased limiting oxygen index (LOI) values and reduced heat release rate by 25%. Moreover, composites of modified fabric showed improvements in static and dynamic mechanical properties. Pickering et al. [35] experimented on aligned short harakeke fiber (New Zealand flax) mats impregnated with epoxy resin. The result showed that these composites were found to possess significantly higher tensile properties at 46% fiber loading, than planar random-oriented short fiber composites, with the values of 136 and 76.2 MPa, respectively. The epoxy resin is a feasible polymer, which has effective strength, good toughness, and appreciable resilience. It has good resistance to moisture and chemical attack. It also has great electrical insulating properties and is devoid of volatile matter [36]. Epoxy resin is broadly used in polymer matrix composite for engineering applications, such as automotive components (e.g., car bumper (Figure 1), door panel, head liner, etc.) [37]. The advantages of epoxy are easy to process at low cost; however, they need to be modified to improve the fracture toughness in order to maintain their demands in automotive applications [38]. Abu Bakar et al. [39], through their study, reported that one of the flaws of natural fibers is poor compatibility with its matrix. Moreover, studies done by Hassan et al. [40] showed that the recyclability of natural fiber within the automotive component had reduced the automotive weight. The use of biocomposite helped in a 25% reduction of vehicle weight, that consequently contributed to saving 39.45 trillion of crude oil [40]. Besides that, this material can be used for the composite frame in electromobility vehicles, as it will reduce energy consumption [25]. Currently, natural fibers are used as fillers to replace glass fiber in polymer composites [16,41,42]. However, using natural fibers as reinforcements encounter fire damage issue. Previous researchers researched to find materials with low flammability and low total emission of heat. Therefore, many fire retardant strategies have been used to improve the flame retardant quality of the composites, in addition to keeping the development safe and environmentally friendly due to the increased use of the natural fiber or polymer composites [43].

The previous studies have shown that certain combinations can increase the flammability of the composites compared to the individual components, whereas others might reduce flammability [45]. The composites had lower fire resistance compared to neat polypropylene, having a shorter time to ignition (TTI) and higher smoke production than neat polypropylene. The samples were burnt for a longer time but the mass-loss rates (MLRs) were lower. Based on the study, when the fiber content was increased above 20%, the characteristics of the composite became similar to lignocellulosic materials. Although the flammability of the composite was also influenced by the construction of the composite, the burning behavior of the composite was largely determined by the properties of the matrix and the reinforcing fibers, and any synergistic or antagonistic effects between them. Moreover, the thickness of the composite could also influence the surface flammability up to a certain limiting value. For the thermally thick samples, the time to ignition (TTI) and duration of the burning were increased when heatwave penetration was less than the thickness of the sample. When the composites were exposed to heat and fire, the bonding between the fiber reinforcement and the matrix polymer was critical, not only for mechanical properties but also for the stability of the composites [43].

The flammability of the fiber reinforcement and the composite as a whole can be reduced by improving the fire retardant of composites strategies [43]. The natural fibers burn very rapidly, which makes retardant treatment for the composite materials very essential for their safe applications [46]. In this study, magnesium hydroxide is used as the fire retardant agent. Magnesium hydroxide is the endothermic flame retardants that work in both gas and condensed phases through endothermic decomposition of releasing non-flammable gases like H_2_O, and CO_2_, which dilute the fuel and cool the polymer. Besides, the lower substrate temperature slows the pyrolysis rate. These materials also lead to protect the underlying polymer. Other examples are aluminum hydroxide, the mixture of huntite and hydromagnesite [47]. Besides, magnesium hydroxide is also a nanosheet that is a significant halogen-free and non-toxic flame retardant.

**Figure 2:**
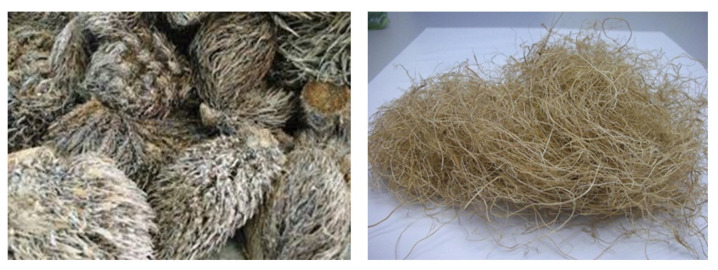
Oil palm empty fruit bunches fiber.

The species *Elemis guineensis Jacq* oil palm in Malaysia, which originates from West Africa, was introduced in Malaysia in the 1860s. Palm oil is a major commodity and is well known in Malaysia [48]. Palm oil plantation is economically important to Malaysia because Malaysia is the second-largest oil palm producer in the world. According to the Malaysian Palm Oil Board (MPOB), the oil palm plantations in Malaysia have increased rapidly to five million from 400 hectares planted in 1920 to 2011. The increasing demand for palm oil is due to the rapid population growth and economic development around the globe. Moreover, in 2013, both Indonesia and Malaysia were the two major palm oil producing countries in the world, with the former producting approximately 31 million tonnes and the latter producing 19.4 million tonnes. The average amount of oil palm fruits produced in Asia was more than 83% of the fruits produced worldwide from 1993 to 2013, which was approximately 159 million tonnes. A total of 22–25% of the fresh oil palm fruit bunch is the empty fruit bunch. Among the various dry fibrous biomass from the oil palm tree, the empty fruit bunch occupies up to 73% of fibers. The production of palm oil leads to the production of vast biomass lignocellulosic residues. These wastes can be in form of oil palm fiber and other lignocellulosic wastes or oil palm empty fruit bunch (OPEFB). Figure 2 shows the oil palm empty fruit bunches’ fiber. The compositions of OPEFB are cellulose, hemicellulose, and lignin. A variety of applications can be produced with the high content of cellulose in OPEFB, including biocomposite films [49].

The OPEFB fiber is seen as the natural acoustic material that has the potential alternative to the existing fibers. Conventionally, the oil palm empty fruit bunch (OPEFB) is mainly used as the burning fuel in boilers to supply electricity for the plantation mills. However, the fibers also have huge potential to be utilized for reinforcement materials in polymer composites. Until recently, early development of research has been carried out on oil palm fiber in relation to its potential as thermal insulating material in the construction of buildings. With the existence of technology to extract the fibers, it is; therefore, feasible to study this waste material’s performance as green and sustainable material [50].

Therefore, in this current work, the investigation was conducted to study the horizontal burning rate where magnesium hydroxide was used in determining its capability as fire-retardant composites. Different percentages of oil palm empty fruit bunches fiber were added with the control of PET yarn and magnesium hydroxide. The objectives of this experiment were to study the horizontal burning rate, tensile strength, and the scanning electron microscopy (SEM) of oil palm empty fruit bunches fiber/PET yarn-reinforced epoxy fire retardant composites. 

## 2. Materials and Method

### 2.1. Materials and Chemicals

The main materials used in this study were oil palm empty fruit bunches (OPEFB) fiber, epoxy polymer, polyester yarn, and magnesium hydroxide. OPEFB fiber was ordered from Innovative Pultrusion Sdn Bhd, Negeri Sembilan. The matrix used in this research was epoxy resin and both the matrix and hardener were supplied by Miracon Sdn Bhd. Table 1 shows the properties of epoxy resin and hardener. The density of the materials used in this research is shown in Table 2.

### 2.2. Fabrication Process

**Figure 3:**
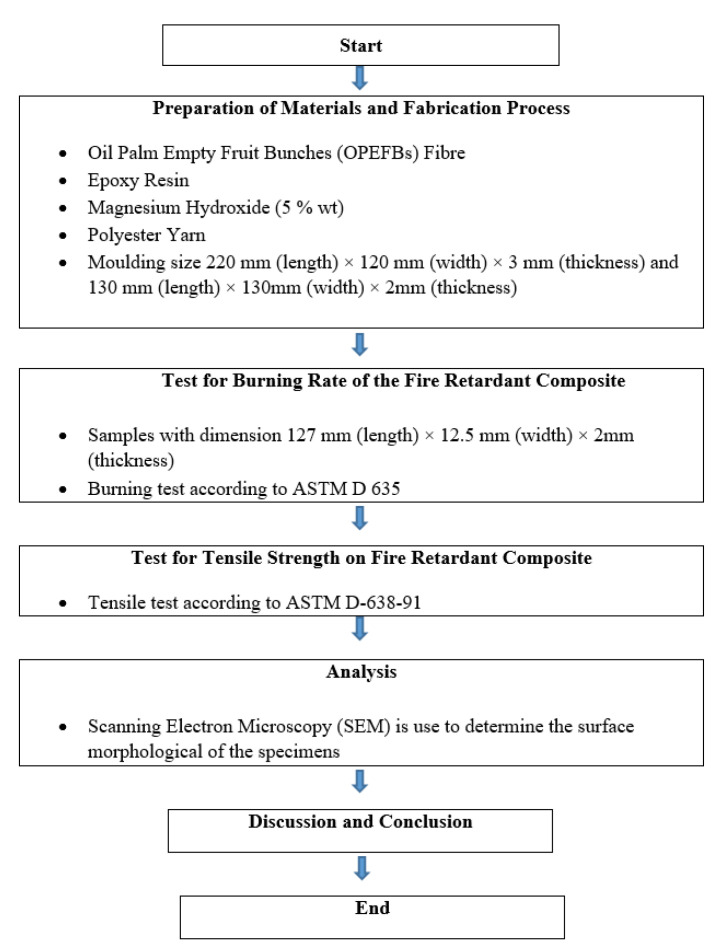
Flowchart of research methodology.

Figure 3 shows the flowchart of the research methodology of this experiment. The composite specimens were prepared by combining the OPEFB fiber with the matrix, which was epoxy resin. This experiment was performed via the hand lay-up technique. The OPEFB fiber reinforced epoxy composites specimens were fabricated with different fiber contents. Table 3 shows different percentages of the fiber used in the fabrication of the specimens.

**Figure 4:**
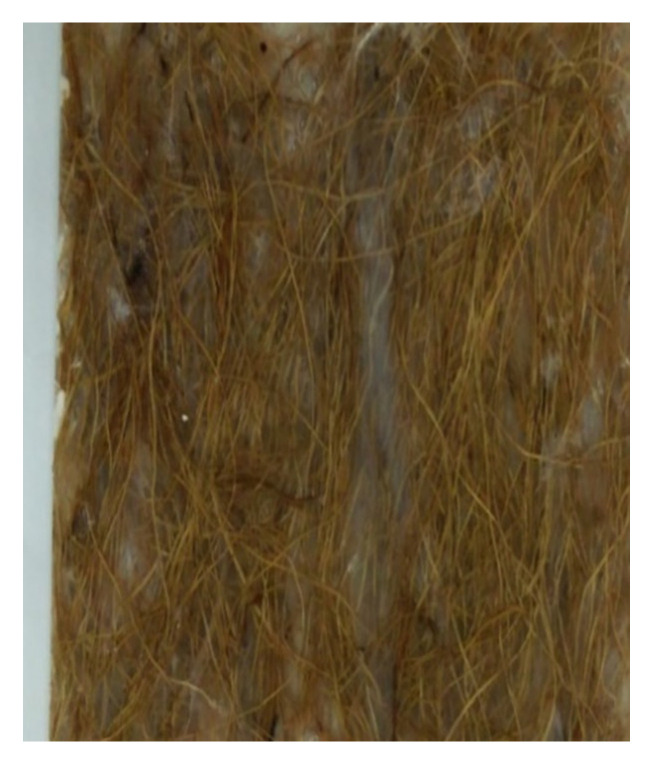
Specimen sample after complete fabrication.

**Figure 5:**
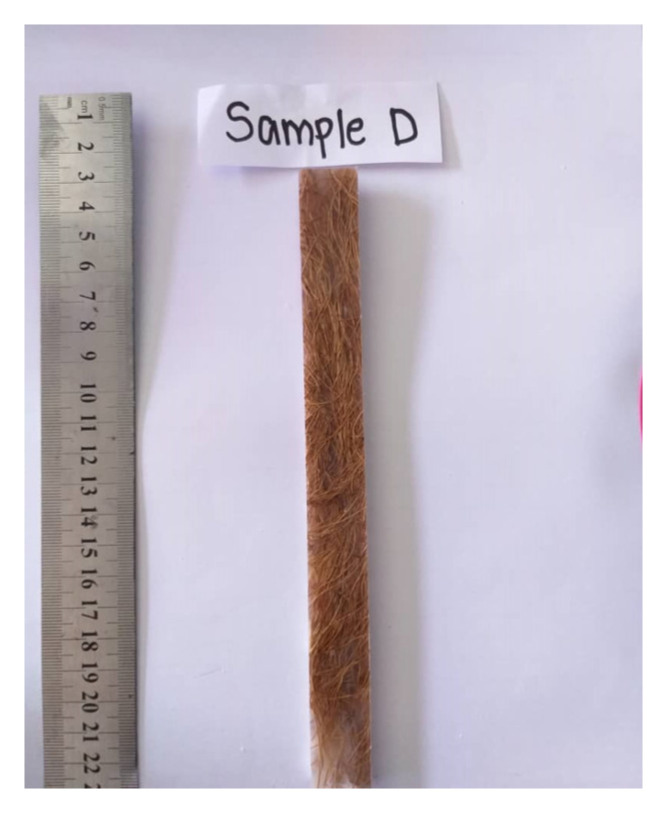
Specimen sample after cutting to 1.8 cm in width.

From the fabrication process, one sample for each parameter produced six specimens with 1.8 cm width. The fiber was combed or straightened and then cut to 22 cm in length before the fabrication. After that, the composites were prepared through the combining of OPEFB fiber, magnesium hydroxide, and PET yarn, with the epoxy resin as a binder. The OPEFB fiber reinforced the epoxy composites specimens were made in four different fiber contents, which were 0%, 20%, 35%, and 50%. The PET yarn was also cut to 22 cm in length. After all materials preparation, the fabrication took place as explained in the following steps. Firstly, the molds were polished by using grease oil which functioned as the releasing agent before the fabrication. Then, the epoxy resin was mixed with a ratio of 2:1 according to its specifications. The hardener reacted as an anhydrate base curing agent. The magnesium hydroxide as a flame retardant agent was added to the epoxy resin after the epoxy resin was uniformly stirred. This mixture of epoxy resin with magnesium hydroxide was stirred using a mechanical stirrer at 1200 rpm for 5 min to prevent the accumulation caused by the chemical reaction. Next, the surface of the mold was wet with epoxy resin. The PET yarn and the OPEFB fiber were put in a random layout in the mold. Then, the mixture was poured into the mold to get the batter surface of the specimens. The composite was covered by a plastic cover before it was compressed by another steel plate to prevent the fiber and PET yarn from attaching to the steel plate during compression work. This molding of the specimen was left for 24 h at room temperature. The specimen was taken out from the mold after it was dried. The specimen was then cut into 6 plates with 1.8 cm width each. Then, the mold was cleaned to be used on the next parameter specimen. For each parameter, the masses of fiber and its binder were calculated to determine the weight of its OPEFB fiber, PET yarn, magnesium hydroxide, and epoxy resin that was needed to fill the mold. Figure 4 and Figure 5 show the sample that was completely cured and cut to 1.8 cm in width, respectively.

### 2.3. Characterization

The burning rate of OPEFB fiber reinforced fire retardant composite was performed through the horizontal burning test. Then, the tensile strength of OPEFB fiber was determined by using the Geotech Testing Machine and followed the determination of defects on the specimen after the tensile test at the ruptured area.

#### 2.3.1. Horizontal Burning Test

**Figure 6:**
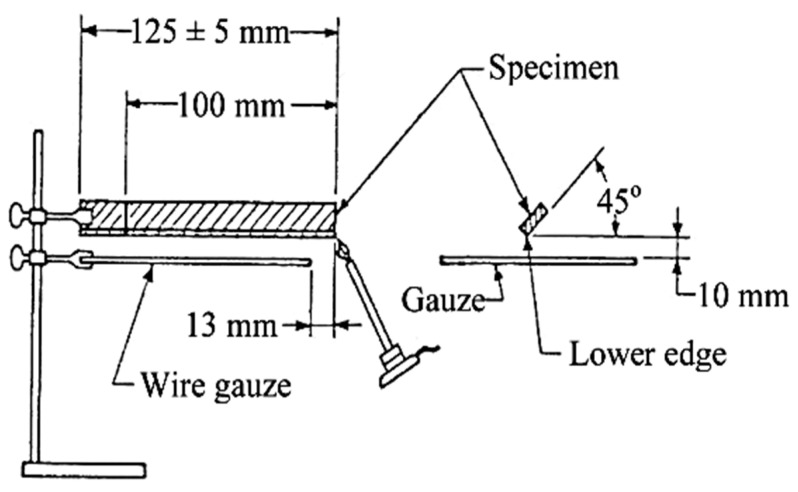
Schematic diagram for burning test setup.

The burning test was conducted to determine the fire resistance of the composites by calculating the burning rate. This test was conducted following the ASTM D 635 standard. The specimens for this burning test were cut to the dimension of 125 × 13 × 2 mm (length × width × thickness). Figure 6 shows the schematic diagram for the burning test setup. The specimen was supported horizontally at one end. Then, the free end was exposed to a specified gas flame for 30 s. After that, the time and extent of burning were measured and reported if the specimen did not burn. The results were recorded and an average burning rate was reported for the specimen if it burnt to the 100 mm mark from the ignited end [51].

#### 2.3.2. Tensile Test

**Figure 7:**
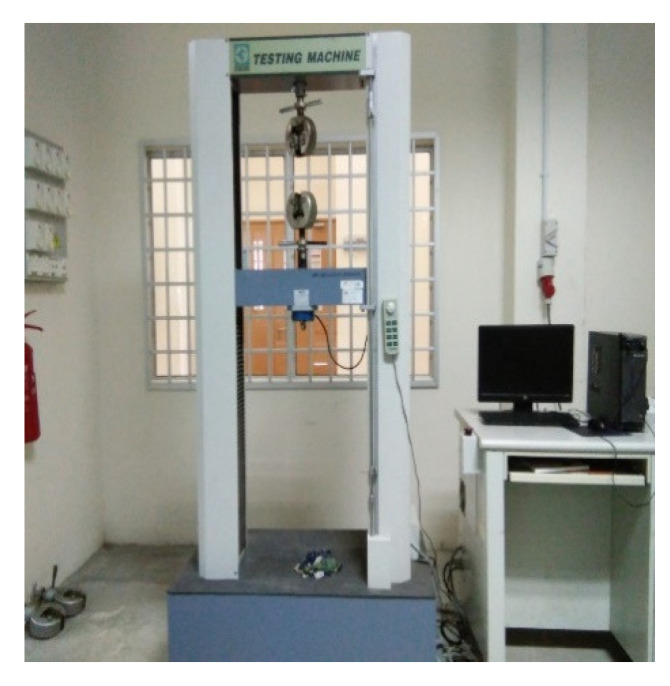
Universal Testing Machine (INSTRON 5556) (Norwood, MA, USA).

A tensile test was conducted to determine the strength of the composites or the mechanical properties of the specimens. This test was set up according to the relevant ASTM D-638-91 standard. The specimens for this test were cut into the dimension 200 × 18 × 3 mm (length × width × thickness). A specimen was put on the top grip jaws and bottom grip jaws of the testing machine and subjected to tensile stress until they fractured. The specimen was ensured not to bend during tightening at the jaws. The grip at the specimen was set up not more than 25 mm. The tensile testing was carried out on a hydraulically operated tensile testing machine as shown in Figure 7. The software U60 was used to set all the necessary conditions and specifications of the specimen for the tensile test. After that, the test was started by clicking the “start” button. After the specimen broke from ultimate stress, it was removed and the tensile tests for other parameters were conducted. The test was carried out on 5 replications using Universal Testing Machine (INSTRON 5556) (Norwood, MA, USA) with a 5 kN load cell; the crosshead speed was maintained at 2 mm/min. From the test, the sample elongation and applied load were measured. Then, the stress and strain were calculated from these values and used to construct the stress–strain graph. From the stress–strain curve, the maximum load, energy, and elastic modulus were determined. The ultimate tensile strength was determined through the maximum load and original cross-sectional area of the specimen. 

#### 2.3.3. Scanning Electron Microscopy (SEM)

**Figure 8:**
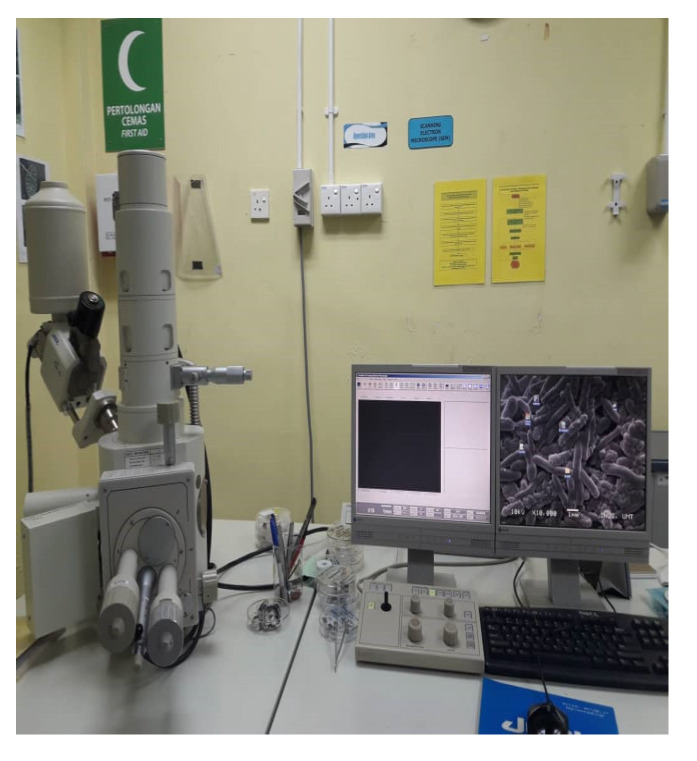
Scanning electron microscope (SEM).

From the tensile test, the fractured specimen was taken to determine the tensile damage that occurred on the surface of the specimen that led to its lower mechanical properties. This test was performed using a scanning electron microscope (SEM), as shown in Figure 8.

The test was started by coating the non-metallic specimen with pure gold by using a sputter coater machine. This step is crucial to make the specimen conductive and ready to be observed under the SEM. The test was started by running the software on the computer to run this experiment. Then, the specimens were put in the beam properly. After that, the “start” button on the software was clicked to run the system. The result was shown when the image of the specimen was on the computer screen. The magnification, image focus, and brightness must be considered to give the best specimen’s defect images. Then, the images were saved and the specimen inside the beam was taken out to change with the other specimens.

## 3. Results and Discussions

### 3.1. Horizontal Burning Test

**Figure 9:**
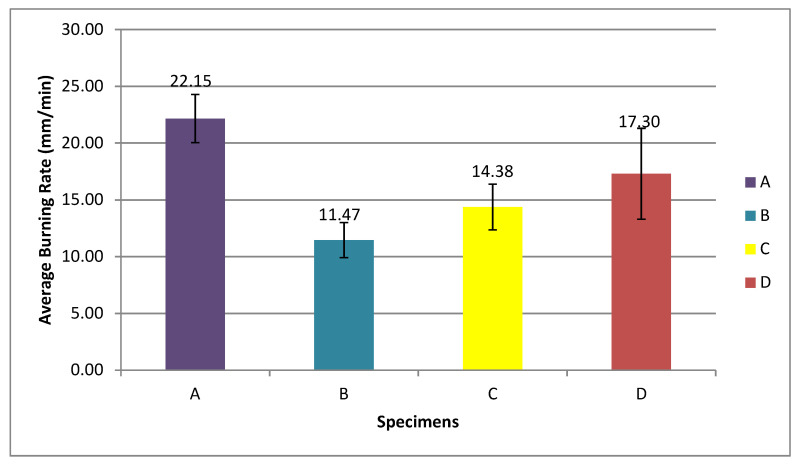
Average burning rate for each specimen.

The dimension of the specimen for this test was 125 (length) × 13 (width) × 2 mm (thickness). From the result of the horizontal burning test for every specimen, the average horizontal burning rates were determined. The calculation showed that the best burning rate was obtained for these OPEFB fiber reinforced fire retardant epoxy composites. In this study, three tests for each parameter or fiber content were performed. This was to make sure that the best average burning rate can be observed. The average burning test for each specimen is illustrated in Figure 9 so that the trend of this average can be clearly seen in the graph.

Equation (1) below shows the calculation for the average burning rate:

(1) $$\mathrm{Average}\;\mathrm{Burning}\;\mathrm{Rate},\;V\;(\mathrm{mm}/\min)\;=\;\frac{\mathrm{Damaged}\;\mathrm{Length},\;L\;\left(\mathrm{mm}\right)}{\mathrm{time},s}.$$

**Figure 10:**
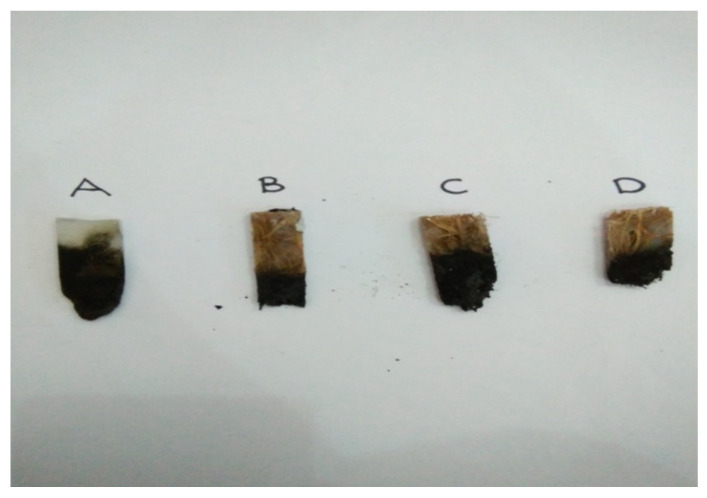
Sample of specimens after the horizontal burning test.

Figure 9 shows the average burning rate for each specimen graph with different fiber volume content in the specimens (20%, 35%, and 50%) that is not linearly increasing. From Figure 9 also, it can be observed that OPEFB fiber with 20% fiber volume content (specimen B) possessed better flammability properties because the lowest average burning rate was 11.47 mm/min, while the average burning rates for fiber volume content of 35% (specimen C) and 50% (specimen D) were 14.38 and 17.30 mm/min, respectively. Specimen A with no fiber content showed the highest average burning rate of 22.15 mm/min. The average burning rate was increased due to the increment of fiber volume content in the specimens. The increased OPEFB fiber volume content resulted in a more inefficient decrement of the composites’ rate of burning [52]. The previous study by Anuar et al. [53] stated that the OPEFB fiber has thick and rough textures. The increase in the OPEFB fiber particle sizes in the composites resulted in a higher burning rate according to the poor dispersion of the larger size OPEFB fillers in the composites as a result of less energy absorption. However, specimen A possessed the highest burning rate because the polymeric matrix exhibited poor flammability behavior. According to [47], the polymer matrix itself depended upon the reinforcements and fillers. Moreover, it had no prominent role in improving the flame resistance of the composites. The PET yarn was melted and tended away from the flame and burned with the smoky flame during the burning test. The magnesium hydroxide acted as the flame retardant to the specimens. According to Alvey et al. [54], styrene solutions of acid-terminated polyester polymers reacted with magnesium oxide and underwent viscosity increment up to the semisolid state. Magnesium hydroxide not only functioned as a flame retardant or smoke suppressant both in the gas phase but also as a reinforcing filler and enhanced the mechanical properties of the matrix [55]. The composition of composites in 20% of fiber volume content (specimen B) was optimum because of the lower burning rate and effectiveness of fire retardant characteristics. Based on [43], when fiber content was increased above 20%, the characteristics of the composite became more similar to the lignocellulosic materials. Figure 10 shows the sample of specimens after the horizontal burning test.

### 3.2. Tensile Properties

**Figure 11:**
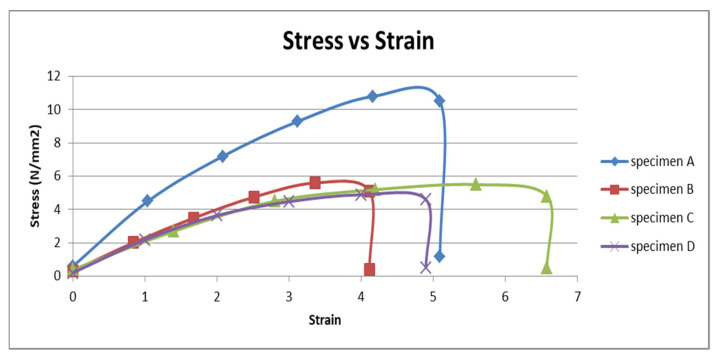
The stress–strain diagram of OPEFB reinforced epoxy composites.

The dimension of the specimens for the tensile test was 200 (length) × 18 (width) × 3 mm (thickness), respectively. In this study, three tests for each parameter of different fiber volume content were performed. The best tensile properties from the three tests were recorded from this study. The yield strength, maximum load, elastic modulus, and energy data were observed in this test. The stress–strain curves of the OPEFB reinforced epoxy composites in Figure 11 were determined after the tensile test. 

Figure 11 shows that the stress–strain curve of OPEFB fiber reinforced epoxy composites with different fiber volume contents, which were specimen A with 0% of fiber, specimen B with 20% of fiber, specimen C with 35% of fiber, and lastly specimen D with 50% of fiber. These curves were linear and followed Hooke’s law. From the curves, it can be observed that the ultimate tensile strength for specimen A (control) was the highest than other specimens containing the fiber. According to Sivaganesan et al. [56], the ultimate tensile strength of oil palm fiber epoxy composites decreased compared to plain epoxy resin. Thus, the higher the fiber volume content in the composite, the lower the ultimate tensile strength of specimens.

**Figure 12:**
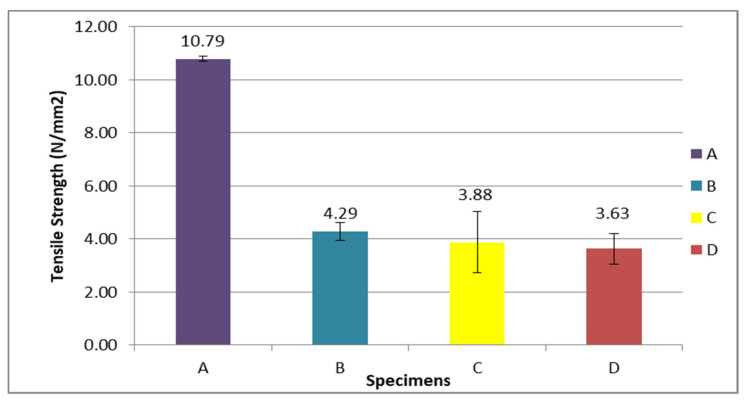
Tensile strength of the OPEFB fiber reinforced epoxy composites.

**Figure 13:**
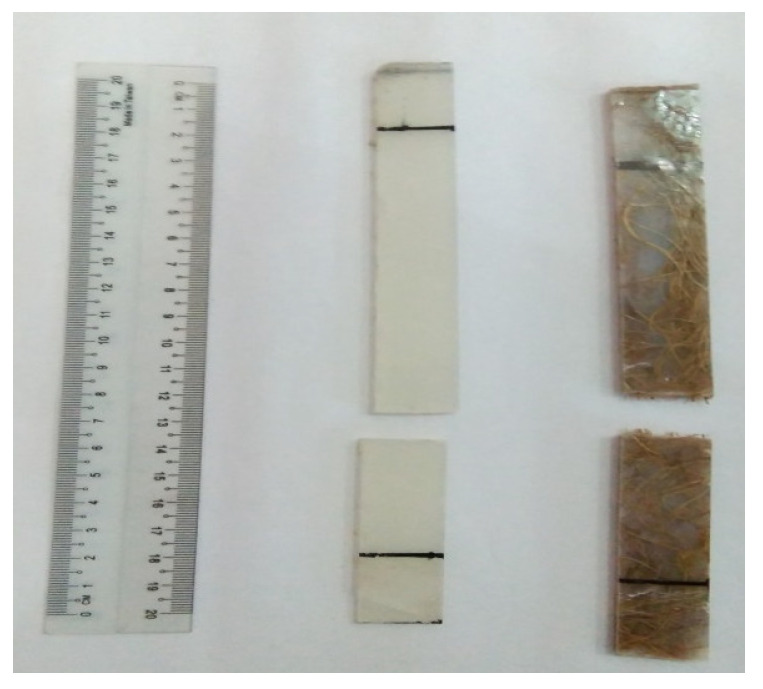
The sample of specimens after the tensile test.

From Figure 12, specimen A had the highest tensile strength from other specimens that contained OPEFB fiber, which was 10.79 N/mm^2^. Specimen B, comprising 20% of OPEFB fiber, was the highest tensile strength among other specimens that consisted of fiber volume content of 4.29 N/mm^2^. While tensile strengths for C and D were 3.88 and 3.63 N/mm^2^, respectively. Specimen A was the highest because the stress during the tensile test was more concentrated in the epoxy matrix. According to Sivaganesan et al. [56], the plain epoxy matrix or control provided better tensile strength, as the stress during the tensile strength was more concentrated in the epoxy matrix rather than the fiber. Besides, the OPEFB fiber was short. The two factors that caused the reduction of stress transfer through the matrix were the incompatibility of polypropylene (PP) and oil palm empty fruit bunch fiber (OPF) and also the irregularity of OPF size [57]. The previous study on short fiber such as OPEFB fiber found that the load applied on the composites structure was then exerted on the matrix surface and transferred to the fiber through the end of the fibers. The capability and capacity that came from the load were mainly carried out by the matrix rather than the fiber [56]. The factor of hydrophilicity or poor adhesion between OPEFB fiber and epoxy matrix could lead to inefficient stress transfer from the epoxy matrix during loading conditions. Therefore, the higher the fiber volume content, the lower the tensile strength. This showed that the increment of fiber that was unable to bind properly with the matrix and had led to the reduction of tensile strength. Besides that, the reduction of tensile strength was also caused by the defects that occurred in the composites during the fabrication. Figure 13 shows the sample of specimens after the tensile test.

### 3.3. Surface Morphology

The surface morphology of all the specimens after the tensile test was conducted by capturing the images of the fractured area. The scanning electron microscopy (SEM) test showed the tensile damage that occurred at the specimen. This tensile damage was related to the defects that always occurred during the manual fabrication of composites known as “lay-up”. The increased defects in the specimen caused the tensile damage in the composites and the reduction of the mechanical properties of the specimen. 

**Figure 14:**
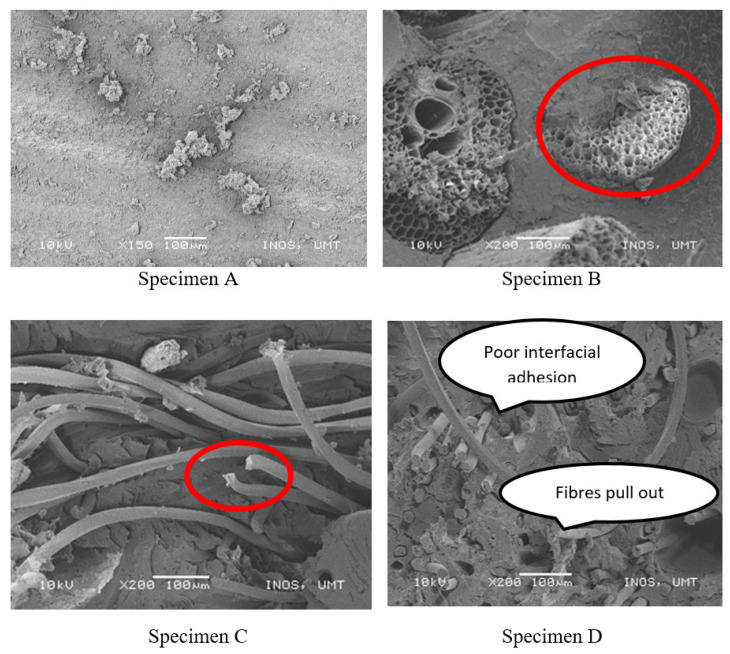
Fibre pull-out and fibre breakage.

Figure 14 shows the surface morphology of specimens A, B, C, and D after the tensile test. It was revealed that the specimen D had the least tensile damage. This led to better mechanical properties, as specimen A possessed the highest tensile strength in the tensile test. This demonstrated that the PET yarn bonded well with the matrix but had the least agglomeration at the surface. 

**Figure 15:**
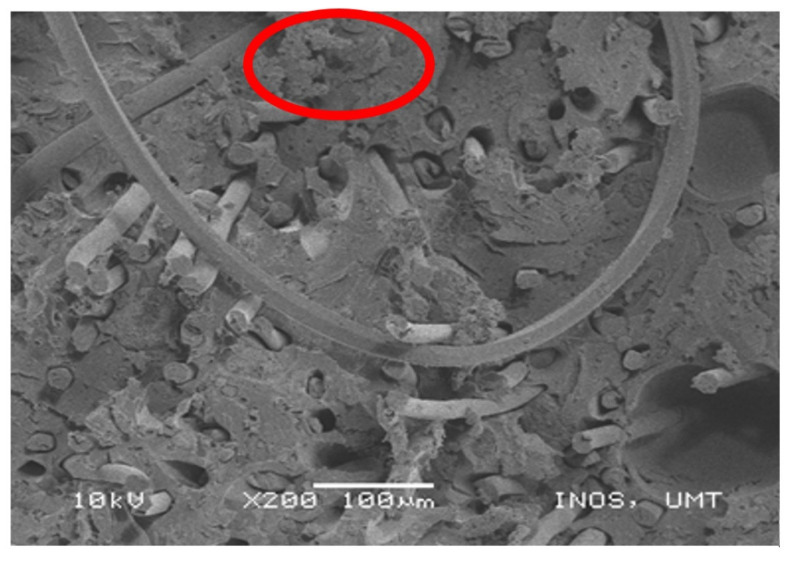
Resin-rich zone.

From Figure 14, the tensile damage was observed, indicated by the fiber pull-outs from the specimens. The defect that occurred on the specimen was poor interfacial adhesions as a result of the hydrophilicity of natural fiber. Therefore, this phenomenon led to poor fiber dispersion and fiber–matrix interfacial adhesion that consequently causing the fiber pull-outs. The fiber–matrix debonding can cause fractures in composites (i.e., fiber pull-out, fiber breakage, and matrix fracture) before the fiber breakage under the applied load [53]. Figure 14 shows that the poor interfacial adhesion of specimens could cause the fiber pull-outs and fiber breakage. Specimen B demonstrated the fiber breakage due to the mechanical force applied to the fiber during the tensile test. Specimen D revealed the fibers pull out caused by poor interfacial adhesion, which resulted in poor mechanical properties of the composites, as explained in the tensile properties section. Figure 15 shows the tensile damage area that might be caused by the content of resin-rich zones. This area will be subjected to greater stress, which is more likely to fail because of weak interfaces in the neighborhood zones. These resin-rich zones were present in the fractured area.

**Figure 16:**
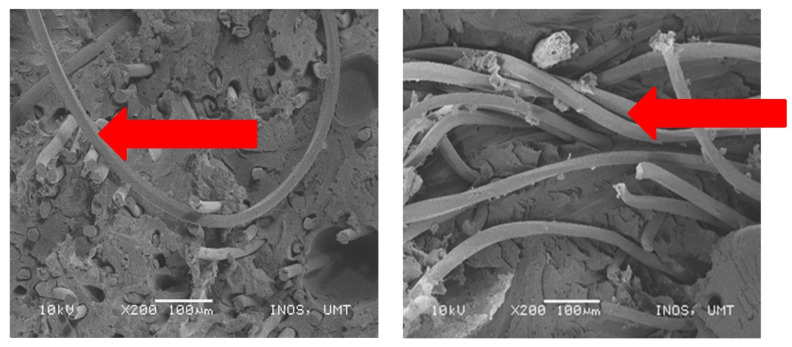
Misalignment of fiber and PET yarn.

After that, the tensile damage that was observed in the specimen was the misalignment of fiber. The results of weak interfacial adhesions of fiber–matrix could result in the misalignment of fibers in the natural fiber reinforced composites. Moreover, this phenomenon might also be caused by poor dispersion of fiber–matrix. Figure 16 below shows the misalignment of fiber and PET yarn that took place in specimens C and D.

## 4. Conclusions

In this study, the OPEFB fiber reinforced epoxy resin was used to fabricate the composite material that has the potential of wide applications in various fields. This is highly contributed by the fiber’s advantages (i.e., low weight, low density, low cost, biodegradable, and good thermal characteristics). Several problems were studied on the current material applications in the maritime field. Among the mainly used material in the maritime field is steel. However, steel encounters corrosion problems and high maintenance costs. In this study, epoxy resin was used because it is a non-corrosive material. Moreover, the natural fibers have their own characteristics similar to OPEFB fiber. The OPEFB fiber is short and short fibers are usually not recommended to be used because of their low mechanical properties. From the horizontal burning test, specimen B (20% of fiber) exhibited the lowest burning rate, which was 11.47 mm/min. Hence, specimen B possessed the best fire retardant properties and 20% of the fiber was concluded to be the optimum fiber composition. In addition, specimens C (35% of fiber) and D (50% of fiber) showed burning rates of 14.38 and 17.30 mm/min, respectively. Specimen A (control) demonstrated the highest average burning rate among other specimens, which was 22.15 mm/min. This horizontal burning test revealed that the increment of fiber volume content in the specimen will give the poor flammability properties of the specimen. After that, the tensile test result exhibited the highest tensile strength observed in specimen A (control), which was 10.79 N/mm^2^. Specimen B (20% of fiber) had the highest tensile strength among the other specimens that contained a fiber volume content of 4.29 N/mm^2^, while specimen C (35% of fiber) and D (50% of fiber) possessed volumes of 3.88 and 3.63 N/mm^2^, respectively. From the study, specimen A had the highest tensile strength because the stress was concentrated more in the epoxy matrix rather than the fiber. This study also demonstrated that the increment of the OPEFB fiber in the composite will lead to a reduction of the tensile strength. The detection of tensile damage in this study through the SEM revealed that specimen D (50% of fiber) exhibited more tensile damage than other specimens that caused the reduction of the tensile strength. Besides, specimen A (control) showed the least tensile damage so it exhibited better tensile strength. The types of defects that were observed in this study consisted of resin-rich zones, misalignment fibers, and regions where resin had poorly wetted the fibers. From this study, the specimens that exhibited more tensile damage were related to the defect that occurred in them and, consequently, revealed the reduction of tensile properties.

## Figures and Tables

**Table 1:** Properties of epoxy resin and hardener.

Property	Epoxy Resin	Hardener
Form	Liquid	Liquid
Density (g/cm^3^)	1.21	1.03
Curing time (h)	24	24
Ratio	2	1

**Table 2:** Densities of materials used.

Material	Density (g/cm^3^)
Oil palm empty fruit bunches (OPEFB)	0.95
PET yarn	1.38
Epoxy	1.21
Magnesium hydroxide (Mg(OH_2_)) powder	1.20

**Table 3:** Specimen fabrication parameters.

Percentage (wt.%)	Specimen Fabrication			
	Specimen A	Specimen B	Specimen C	Specimen D
**Epoxy + Hardener**	90	70	55	40
**PET Yarn**	5	5	5	5
**Magnesium Hydroxide**	5	5	5	5
**OPEFB Fibre**	0	20	35	50

## Data Availability

The data presented in this study are available on request from the corresponding author.
